# Hydrogel-Dependent Angiogenic Sprouting in the Ex Vivo Aortic Ring Assay: A Comparative Functional Approach for Biomaterial Evaluation

**DOI:** 10.3390/jfb17090425

**Published:** 2026-08-24

**Authors:** Lisa Götz, Leyla Dogan, Philipp Wörsdörfer, Nathaly A. Chicaiza-Cabezas, Süleyman Ergün, Jürgen Groll, Florian Kleefeldt

**Affiliations:** 1 Institute of Anatomy and Cell Biology, University of Würzburg, 97070 Würzburg, Germany; 2 Department of Functional Materials for Medicine and Dentistry, Institute for Functional Materials and Biofabrication (IFB), University of Würzburg, 97070 Würzburg, Germany; 3 Atlas University Research Center (ARC), Istanbul Atlas University, Istanbul 34403, Turkey

**Keywords:** aortic ring assay, biomaterial evaluation, angiogenesis, vascular sprouting, tissue engineering

## Abstract

Insufficient vascularization remains a major limitation in tissue engineering, restricting the survival and maturation of larger bioengineered constructs. While candidate hydrogels are commonly characterized with regard to physicochemical properties, gelation behavior, mechanical performance, and cytocompatibility, simple functional assays that assess their capacity to support vascular sprouting are less frequently integrated into early-stage biomaterial evaluation. Here, we investigated the established ex vivo aortic ring assay (ARA) as an exploratory functional approach for the initial comparison of selected hydrogel formulations. Murine aortic rings were embedded in collagen I (Col I), alginate (Alg), or gelatin methacryloyl (GelMA) and cultured under control conditions or with vascular endothelial growth factor A (VEGF-A) stimulation. These hydrogels were intentionally selected as a proof-of-concept panel of representative, non-equivalent material classes with distinct expected cell-interactive properties. After five days, Col I supported robust capillary-like outgrowth that was further enhanced by VEGF-A, whereas the tested GelMA formulation supported only limited cellular migration and the tested unmodified Alg formulation showed no detectable sprouting under the conditions examined. Cluster of differentiation 31 (CD31) immunostaining supported the presence of an endothelial component within the Col I-supported sprouting structures. These findings demonstrate that the ARA can detect pronounced formulation-dependent differences among the specific hydrogels tested using straightforward morphological and immunostaining readouts. Within the scope of the formulations tested, these findings support the ARA as a complementary functional readout alongside conventional biomaterial characterization before more complex tissue engineering or biofabrication studies are performed.

## 1. Introduction

The engineering of thick and metabolically active tissues remains limited by the difficulty of establishing functional vascularization [1,2]. Tissue engineering combines cells, biomaterial matrices, biochemical cues, and fabrication strategies to generate tissue substitutes and experimental tissue models. These are relevant for regenerative medicine, transplantation research, and disease modeling [3]. For these purposes, the selection of suitable biomaterials is central, as their structural, mechanical, and biological properties influence cell survival, tissue organization, and functional maturation of engineered constructs [4]. Three-dimensional (3D) bioprinting and related biofabrication technologies have enabled the precise and reproducible assembly of increasingly complex tissue architectures [2,3]. However, because physicochemical characterization alone cannot fully predict whether a hydrogel also supports vascular sprouting, candidate materials should be evaluated using simple and robust functional assays before integration into more complex tissue engineering or biofabrication workflows [5,6].

Strategies to promote vascularization include angiogenic stimulation, in vitro pre-vascularization, incorporation of vascular cells, and the use of extracellular matrix-derived or engineered materials [7]. In this context, hydrogels have gained particular importance since they provide three-dimensional matrices for cells and can be tuned with regard to gelation behavior, mechanical performance, degradation properties, and biochemical functionality [8]. However, designing hydrogels that effectively support angiogenic sprouting remains challenging, as material performance depends on the balance between biocompatibility, mechanical properties, degradation kinetics, cell–matrix interactions, and the availability or release of angiogenic factors such as vascular endothelial growth factor A (VEGF-A) or fibroblast growth factor (FGF) [5,6]. In addition, hydrogel performance is influenced by tissue-specific requirements, host integration, and immune responses [8]. Thus, conventional physicochemical and cytocompatibility testing should be complemented by functional biological assays that directly assess whether candidate matrices support vascular outgrowth [9,10].

The aortic ring assay (ARA) is an established and biomimetic ex vivo assay that retains a multicellular vascular tissue context and enables the analysis of angiogenic sprouting and vascular network formation [10,11]. Angiogenesis is a coordinated process involving endothelial cell activation, extracellular matrix remodeling, directed migration, proliferation, sprout extension, and the contribution of perivascular and stromal cell populations [12,13]. These events are linked to morphogenesis-associated processes, including cell–matrix interactions, multicellular organization, and tissue remodeling [14]. Thus, the ARA does not replace detailed mechanistic analyses or physicochemical material characterization but provides a practical and relatively simple biological readout to assess whether a matrix environment is permissive for vascular sprouting and early sprout organization [10,11].

In this study, we investigated the established ARA as a comparative functional workflow for hydrogel evaluation under identical experimental conditions. Based on their widespread use in tissue engineering and their expected differences in cell adhesive and sprouting permissive properties, we selected collagen I (Col I), gelatin methacryloyl (GelMA), and alginate (Alg) as a proof-of-concept matrix panel [11,15,16]. The formulations were intentionally selected as representative, non-equivalent hydrogels rather than as mechanically or biochemically matched materials. The specific conceptual contribution of this study is the application of the established ARA to directly compare distinct hydrogel formulations within a single experimental workflow under basal and VEGF-A-stimulated conditions. Col I served as an extracellular matrix-derived, sprouting-permissive reference matrix that is frequently used in ARA [11]. Alg was included as a widely used hydrogel with favorable handling and gelation properties but limited intrinsic support for cell adhesion in its unmodified form [16,17]. GelMA was chosen as an engineered gelatin-based hydrogel with relevance for tissue engineering and downstream biofabrication workflows [15,18]. By quantifying angiogenic outgrowth, we assessed whether the ARA can reveal formulation-dependent differences in the integrated biological performance of the tested hydrogels without attributing these responses to individual physicochemical material properties.

## 2. Methods

### 2.1. Animals

C57BL/6J mice (Jackson Laboratory, Bar Harbor, ME, USA) were kept under specified pathogen-free conditions on a 12:12 h light/dark cycle and had ad libitum access to standard chow and autoclaved water (Center for Experimental Molecular Medicine, University of Würzburg, Würzburg, Germany). No experimental treatment or intervention was performed prior to euthanasia, and the animals were used exclusively for organ and tissue collection. The use of animals for this purpose was notified to the competent local authority, the Government of Lower Franconia (Regierung von Unterfranken, Würzburg, Germany), and was conducted in accordance with German animal welfare regulations and institutional requirements. As the animals were used solely for tissue collection without preceding experimental procedures, no separate animal experiment approval number was assigned.

### 2.2. Hydrogel Preparation

Rat type I collagen (Merck, Darmstadt, Germany; 08-115; 1 mg/mL; pH 7.0), sterile 2% (*w*/*v*) sodium alginate, and porcine gelatin methacryloyl (GelMA) were used as hydrogel matrices.

To prepare the Alg solution, sodium alginate (Merck; PH132S2) was sterilized under UV light for 1 h and dissolved in phosphate-buffered saline (PBS) by stirring overnight at 37 °C. The prepared hydrogel was stored under sterile conditions at 4 °C until use. Prior to use, the Alg solution was equilibrated to 37 °C.

GelMA was synthesized as described by Loessner et al. [18]. Briefly, 10% (*w*/*v*) gelatin from porcine skin (Merck; G2500) was dissolved in PBS and reacted with 60% (*w*/*w*) methacrylic anhydride while stirring vigorously at 37 °C for 1 h. Unreacted methacrylic anhydride was removed via centrifugation (3500× *g*, 3 min, room temperature [RT]). The GelMA-containing supernatant was diluted with two volumes of prewarmed (40 °C) Milli-Q water, dialyzed (molecular weight cut-off 3.5 kDa, 3 days), and pH was adjusted to 7.4. After sterilization using 0.2 µm syringe filters, GelMA was lyophilized and stored protected from light at −20 °C. For use, GelMA was dissolved at 5% (*w*/*w*) in PBS under magnetic stirring (200 rpm, 47 °C) and mixed with 0.1% (*w*/*v*) lithium phenyl-2,4,6-trimethylbenzoylphosphinat (Merck; 900889). Once fully dissolved, the mixture was maintained in a water bath at 37 °C.

### 2.3. Aortic Ring Assay

The ARA was performed as previously described [19]. Eight-week-old mice were euthanized by cervical dislocation and fixed back-down on a dissecting board. The ventral skin was sterilized with 70% (*v*/*v*) ethanol and removed. The thoracic cavity was opened by incision of the diaphragm and bilateral cutting of the ribs to expose the heart. Opening of the right atrium enabled venous drainage, and PBS perfusion was performed via the left ventricle until the intravascular blood was cleared.

After identification of the aorta, perivascular fat tissue was carefully removed in situ under a stereomicroscope using fine forceps and spring scissors. Care was taken to avoid damage to the adventitia while ensuring complete removal of contaminating tissue, potentially including progenitor cell populations that may affect experimental outcomes [19]. The thoracic aorta was isolated by transecting it above the diaphragm, gently mobilizing the vessel to detach intercostal branches, and cutting the proximal end at the origin of the left subclavian artery. The dissected aorta was transferred to ice-cold PBS and kept on ice until further processing. The vessel was additionally rinsed with PBS to ensure complete clearance of intraluminal blood and sectioned into ring segments of 1 mm thickness. After overnight incubation in Opti-MEM (Thermo Fisher, Waltham, MA, USA) at 37 °C and 5% CO_2_, aortic ring segments were embedded in hydrogel. To this end, 80 µL of hydrogel was dispensed into each well of a 96-well plate and aortic rings were embedded ensuring an upright orientation of the lumen to allow direct visualization and quantification of the sprouting. Gel solidification was induced according to hydrogel type: Col I matrices were polymerized by incubation at 37 °C and 5% CO_2_ for 1 h, Alg gels were crosslinked using 20 mM calcium chloride (Merck; EC: 233-140-8) for 20 min, and GelMA was exposed to 405 nm light (54 mJ/cm^2^, 10 s) for crosslinking. Aortic rings were cultured in Dulbecco’s modified Eagle medium (DMEM)/10% fetal calf serum (FCS) (Thermo Fisher/Capricorn, Ebsdorfergrund, Germany) with or without supplementation with vascular endothelial growth factor A (VEGF-A; Thermo Fisher; 50 ng/mL) at 37 °C and 5% CO_2_ and culture medium was replaced every third day. Images were acquired on day 5 and aortic rings were fixed on day 7 for immunostaining.

For each experimental condition, aortic rings from three individual animals were used. Six rings per animal and condition were analyzed as technical replicates. For statistical analysis, technical replicates from each animal were averaged, and animal means were used as biological replicates.

### 2.4. Imaging and Quantification

Phase-contrast images were acquired using a Leica DFC 3000 G microscope (Leica Microsystems, Wetzlar, Germany). Angiogenic outgrowth was quantified manually using ImageJ (National Institutes of Health, Bethesda, MD, USA; version 1.54p). The total two-dimensional area comprising the aortic ring and surrounding cellular outgrowth was outlined. The area of the aortic ring itself was then subtracted from this total area in order to calculate the effective sprouting area. This parameter was selected as a simple and rapid readout for the initial comparison of whether different biomaterial formulations support angiogenic outgrowth under defined conditions. It assesses overall outgrowth but does not provide a detailed characterization of sprout number, length, branching, or vascular network architecture.

### 2.5. Immunostaining

Cluster of differentiation 31 (CD31) immunostaining was performed to assess the presence of an endothelial component within the organized sprouting structures. Because these structures were only observed in Col I matrices but not in GelMA or Alg under the tested conditions, staining was performed only for the Col I matrix. Col I-embedded aortic rings were fixed in 4% paraformaldehyde for 24 h at 4 °C in the 96-well plate. After permeabilization (0.1% Triton X-100; 15 min, RT), whole-mount immunofluorescence staining for the endothelial marker CD31 (R&D Systems, Minneapolis, MN, USA; AF3628; 1:50; 24 h, 4 °C) was performed within the wells according to previously described protocols [20]. Nuclei were stained with 4,6-diamidino-2-phenylindole (DAPI; Merck; 10236276001; 1:10,000; 10 min, RT). The Cy5-labeled secondary antibody was purchased from Dianova^TM^ (Biozol Diagnostica Vertrieb GmbH, Hamburg, Germany; 705-175-147; 1:250; 1 h, RT). Aortic rings were extracted from the Col I matrix and placed on a slide. They were then mounted with Mowiol and flattened under a coverslip. The samples were analyzed using a BZ-9000 fluorescence microscope (Keyence Corporation, Osaka, Japan).

### 2.6. Statistical Analyses

Statistical analyses were performed using OriginPro software (OriginLab Corporation, Northampton, MA, USA; version 2021b). For each experimental condition, six aortic rings from each of three individual animals were analyzed as technical replicates. Technical replicates from each animal were averaged, and the resulting animal means were used as independent biological replicates (*n* = 3 animals per condition). Data are presented as mean ± standard error of the mean (SEM). Comparisons between two groups were performed using paired Student’s *t*-test, whereas comparisons among three groups were analyzed using one-way analysis of variance (ANOVA), followed by Tukey’s post hoc multiple-comparison test. Statistical significance was defined as *p* ≤ 0.05.

## 3. Results

### 3.1. Hydrogel-Dependent Differences in Angiogenic Sprouting

**Figure 1:**
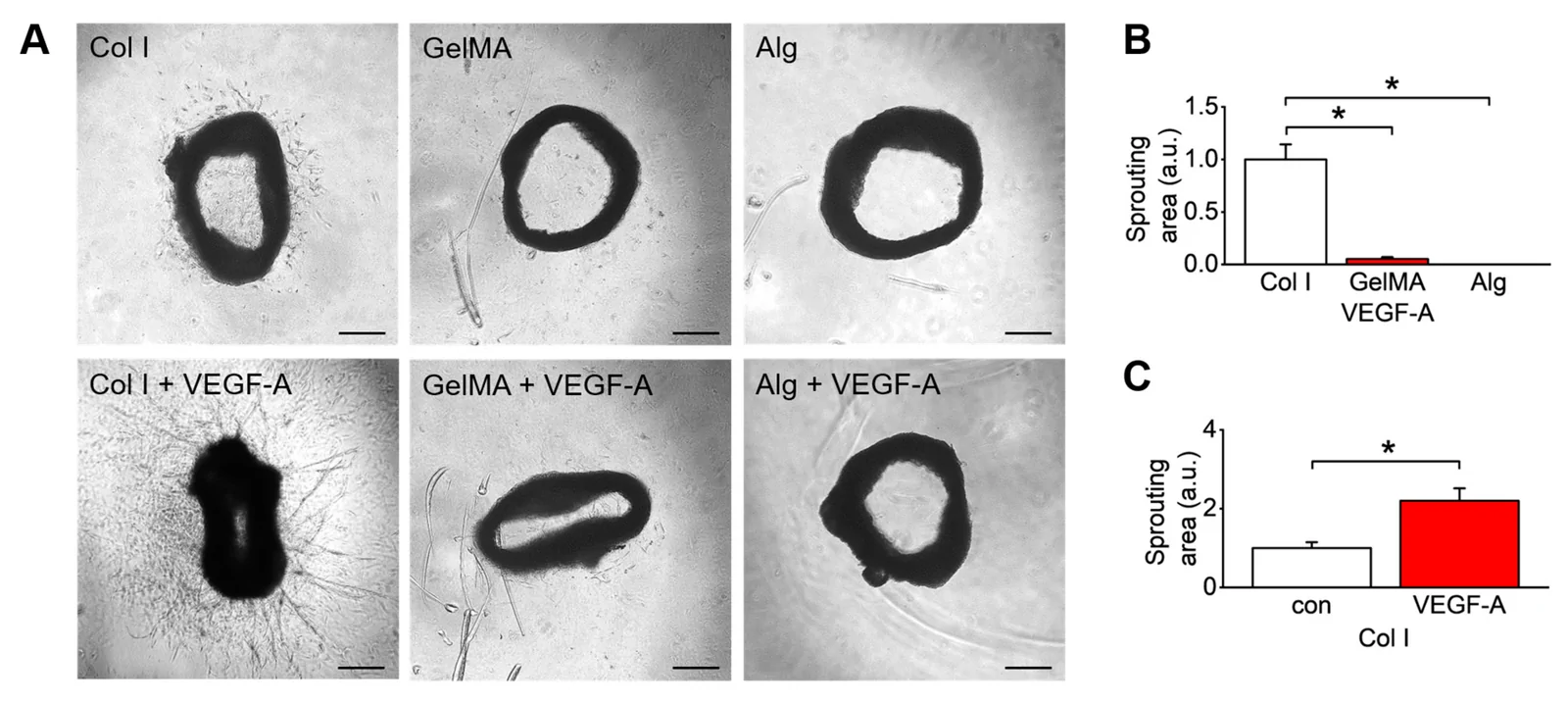
Hydrogel-dependent angiogenic sprouting of aortic rings. (**A**) Representative micrographs of murine aortic rings embedded in collagen I (Col I), gelatin methacryloyl (GelMA), or alginate (Alg) cultured for 5 days under control or VEGF-A (50 ng/mL) conditions. Col I supported angiogenic sprouting, which was enhanced by VEGF-A, resulting in elongated and branched sprouting structures. The tested GelMA formulation showed only limited spreading of individual cells, while the tested unmodified Alg formulation did not support cellular outgrowth under the conditions tested. (**B**) Quantification of effective sprouting area (total area comprising the aortic ring and cellular outgrowth minus the aortic ring area) shows significantly greater outgrowth in Col I compared to GelMA and Alg. (**C**) VEGF-A increased sprouting in collagen-embedded aortic rings. Images were acquired using a 5× objective. Scale bars: 250 µm. * *p* ≤ 0.05. a.u., arbitrary units.

To evaluate whether the ARA detects hydrogel-dependent differences in angiogenic sprouting, aortic rings were embedded in Col I, GelMA or Alg and cultured under either control conditions or in the presence of VEGF-A. Five days after embedding, cellular sprouting was observed in Col I samples. Under control conditions, Col I-embedded rings exhibited modest sprouting, characterized by migration of single cells. In contrast, VEGF-A treatment markedly enhanced cellular extension, resulting in elongated and branched sprouting structures consistent with increased sprout formation and spatial organization in response to pro-angiogenic stimulation (Figure 1A). In contrast, the GelMA formulation tested in this study supported only limited cellular migration under the conditions tested. Even under VEGF-A stimulation, only sparse migration of individual cells was detected within the hydrogel, without the formation of elongated sprouting structures. Notably, aortic rings embedded in the tested unmodified Alg formulation showed no cellular outgrowth or sprouting under either control or VEGF-A-treated conditions, indicating that this specific Alg formulation did not support detectable vascular sprouting under the experimental conditions used.

Quantitative analysis of the effective sprouting area was used as a straightforward morphological readout of angiogenic outgrowth. This analysis revealed significantly greater vascular outgrowth in Col I compared with the specific GelMA and Alg formulations tested (Figure 1B). VEGF-A treatment further increased the sprouting area in Col I hydrogels whereas no comparable increase was detected in GelMA or Alg under the tested conditions (Figure 1C).

### 3.2. CD31 Expression in Collagen-Supported Angiogenic Sprouts

**Figure 2:**
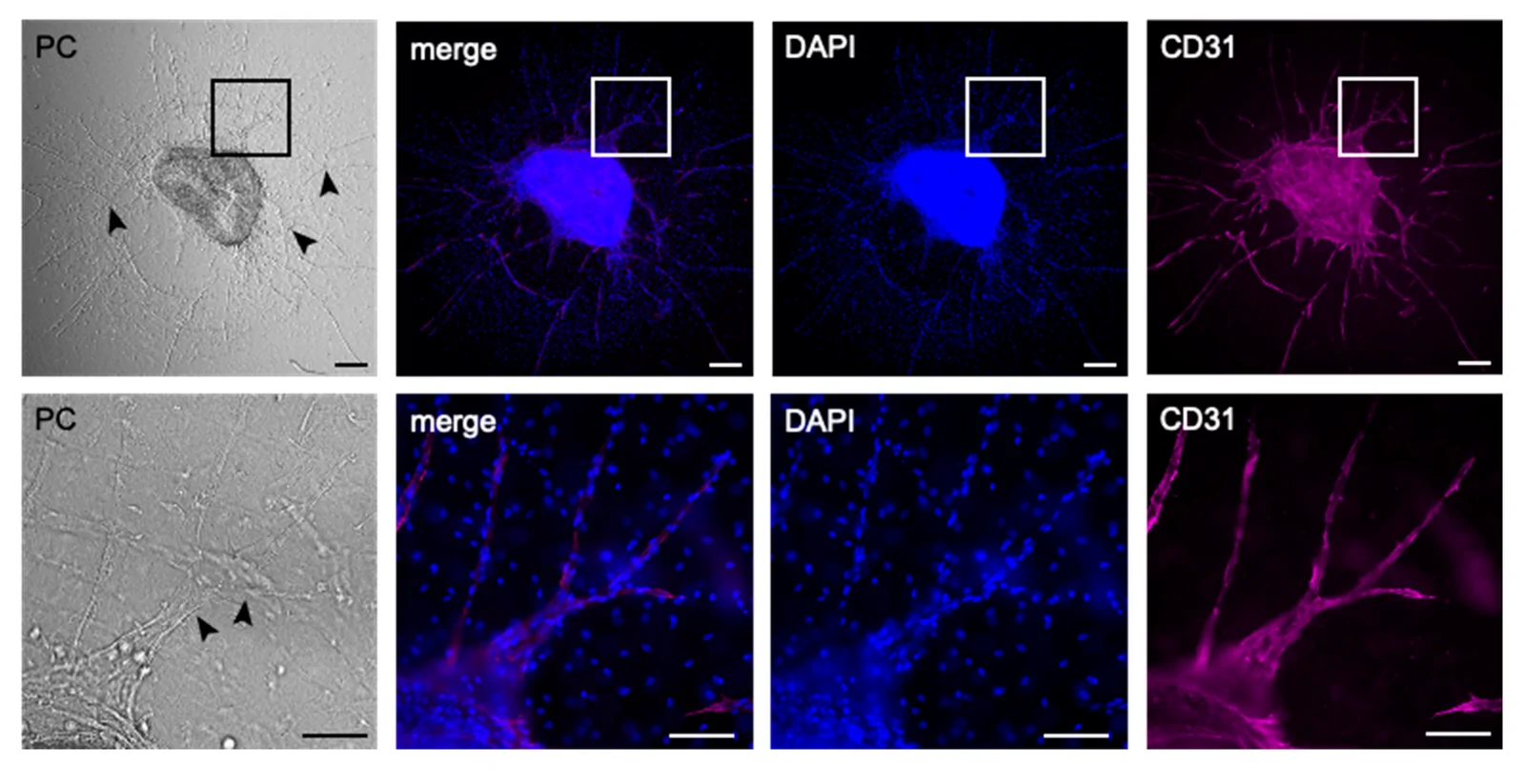
CD31 expression in collagen-supported sprouting structures. Representative phase-contrast (PC, **left** panels) and immunofluorescence images (**right** panels) of aortic rings embedded in Col I and cultured for 7 days in the presence of VEGF-A (50 ng/mL). PC panel shows elongated and branched capillary-like structures (black arrowheads). Immunofluorescence staining reveals CD31 expression along the sprouting branches, supporting the presence of an endothelial component within the outgrowth. The comparison of Col I control and VEGF-A-stimulated conditions is presented in Figure 1. Nuclei were counterstained with DAPI. The DAPI panels show nuclear counterstaining alone, whereas the merge panels show the overlay of the DAPI and CD31 fluorescence signals. Overview images were acquired using a 4× objective, and higher-magnification images were acquired using a 20× objective. Scale bars: overview images, 250 µm; higher-magnification images, 100 µm.

As organized capillary-like sprouting structures were observed in Col I matrices but not in GelMA or Alg under the tested conditions, CD31 immunostaining was focused on Col I-supported sprouts. Figure 2 shows a representative example of the CD31-positive sprouting structures observed under VEGF-A stimulation. The comparison between Col I control and VEGF-A-stimulated conditions, including the quantitative analysis of the complete replicated dataset, is presented in Figure 1. Whole-mount immunofluorescence staining revealed CD31 immunoreactivity along elongated and branched structures, supporting the presence of an endothelial component within the outgrowth.

## 4. Discussion

Recent advances in tissue engineering have enabled increasingly precise control over tissue architecture, cellular composition, and matrix design [3]. However, the generation of functional vascular networks remains a major challenge that limits the survival, integration, and maturation of larger engineered constructs [1,2]. In this context, candidate hydrogels are commonly optimized with regard to physicochemical and processing-related parameters such as gelation behavior, mechanical stability, degradation properties, handling, and cytocompatibility [8,15]. Although these parameters are essential for material development, they do not necessarily predict whether a matrix supports vascular sprouting in a multicellular tissue context [5,6]. Functional biological assays are therefore needed to complement conventional biomaterial characterization and to support early-stage material selection and refinement [9,10].

In the present study, we show that the established ex vivo ARA can be applied as a comparative functional workflow to reveal hydrogel-dependent differences in angiogenic sprouting under identical experimental conditions [10,11]. Col I supported robust cellular outgrowth and VEGF-A-responsive elongated and branched sprouting structures, whereas the tested GelMA formulation permitted only limited cellular migration and the tested Alg formulation did not support detectable outgrowth under the conditions examined. CD31 immunostaining supported the presence of an endothelial component within the Col I-supported sprouts. By assessing both overall sprouting and VEGF-A responsiveness in a direct side-by-side comparison, the assay distinguished formulation-dependent angiogenic responses within a single experimental workflow. Thus, the ARA provided a straightforward biological readout to distinguish matrices that support aortic ring-derived vascular sprouting from matrices that are non- or only weakly permissive under the tested conditions.

The three hydrogels used in this study were intentionally selected as a focused proof-of-concept panel of representative, non-equivalent material classes. Their distinct cell-interactive properties were expected to result in different sprouting responses, thereby allowing us to assess whether the ARA can detect formulation-dependent differences under identical experimental conditions. The study was not designed to compare mechanically or biochemically matched hydrogel systems or to establish a general superiority of Col I over GelMA or Alg. Col I was used as an extracellular matrix-derived reference material that is frequently applied in the ARA [11]. Alg was included because it is widely used in tissue engineering due to its favorable handling and gelation properties [16]. At the same time, its unmodified form lacks intrinsic cell adhesive ligands and was therefore expected to provide a low permissive matrix environment for vascular sprouting [16,17]. As a third matrix, GelMA was included as an engineered gelatin-based hydrogel with relevance for tissue engineering and downstream biofabrication workflows [15]. Its biological performance depends strongly on formulation-specific parameters, including the degree of methacrylation and the applied crosslinking conditions [15,18]. Accordingly, the present findings apply only to the formulations and processing conditions examined and should not be generalized to GelMA or Alg hydrogels as material classes. Moreover, because the three formulations were not comparatively characterized with respect to stiffness, swelling behavior, degradation, pore architecture, or rheological properties, the observed differences cannot be attributed to material chemistry or to any individual physical property. These parameters are known to influence angiogenic sprouting [5,6,21]. The ARA therefore assesses the biological response to each material formulation under the conditions tested, rather than identifying the individual material property responsible for that response. In this context, the aim of the present study was to investigate whether the ARA can provide a simple comparative biological readout for the specific hydrogel formulations tested.

Conventional cytocompatibility assays, such as simple viability tests, are valuable for detecting overt cytotoxic effects of candidate matrices [9]. However, they provide only limited information on coordinated processes in a multicellular environment, such as cellular outgrowth, sprout formation, and multicellular organization [9,10]. By preserving the native architecture of the vessel wall and maintaining a multicellular tissue context, the ARA provides a functional biological readout that can complement conventional cytocompatibility assays [10,11,19]. In the present study, we focused on sprouting area as an initial readout, complemented by VEGF-A responsiveness and CD31-based assessment of an endothelial component within the outgrowths. These readouts are simple, cost-effective and broadly implementable [10,11]. Our findings support the feasibility of using the ARA for the initial functional comparison of selected hydrogel formulations.

Within a staged biomaterial development pipeline, endothelial tube formation assays provide a rapid, cost-effective, and relatively high-throughput assessment of endothelial network formation [7,22]. However, they lack the multicellular context of native vascular tissue [22]. The ARA preserves interactions among endothelial, perivascular, stromal, and resident immune cells, although at lower throughput [11,19]. Microfluidic vascular models additionally enable controlled perfusion, biochemical gradients, and detailed mechanistic analyses but require greater technical expertise and resources [22,23]. In vivo angiogenesis models capture systemic responses and tissue integration more comprehensively but are more time-consuming, costly, and ethically demanding [7,22]. The ARA may therefore serve as an intermediate functional evaluation step between simple in vitro screening and more complex microfluidic or in vivo validation [22].

Several limitations of this study should be considered. First, we tested only three hydrogel matrices. Moreover, the hydrogels were not comparatively characterized with respect to their mechanical, rheological, swelling, degradation, or structural properties. This represents a major limitation because these parameters can influence vascular sprouting and prevent attribution of the observed differences to a specific physical property or to material chemistry alone [5,6,21]. Second, the analysis focused on vascular sprouting using two-dimensional sprouting area, VEGF-A responsiveness, and CD31 immunostaining. Although sprouting area provides a practical measure of overall angiogenic outgrowth, it does not capture parameters such as sprout number, length, or branching density [10]. The present study therefore does not provide a detailed characterization of vascular network morphology. Moreover, CD31 staining alone cannot conclusively identify all cells within the outgrowth or demonstrate the formation of mature vascular structures. Finally, the ARA is a murine ex vivo assay. Although it mimics key aspects of angiogenic sprouting in a multicellular tissue context, it does not fully recapitulate human tissue integration, perfusion, or host immune responses [11,22]. Furthermore, the assay does not test the mechanical and processing limitations that are also central for subsequent biofabrication workflows [3,15]. Therefore, the ARA is not intended to replace physicochemical, rheological, cytocompatibility, or application-specific testing. Rather, our results indicate that the ARA may provide a complementary biological readout for the initial evaluation of selected hydrogel formulations under defined experimental conditions.

Overall, our findings support the feasibility of using the ex vivo ARA for the initial functional comparison of the specific hydrogel formulations tested. By revealing formulation-dependent differences in vascular sprouting, the assay may complement conventional biomaterial characterization and inform the subsequent evaluation or refinement of candidate hydrogels. Broader validation across additional biomaterials, formulations, and physicochemical conditions will be required before this approach can be generalized.

## Data Availability

The raw data supporting the conclusions of this article will be made available by the authors on request.

## Abbreviations

Alg	alginate
ANOVA	analysis of variance
ARA	aortic ring assay
CD31	cluster of differentiation 31
Col I	collagen I
DAPI	4,6-diamidino-2-phenylindole
DMEM	Dulbecco’s modified Eagle medium
FCS	fetal calf serum
FGF	fibroblast growth factor
GelMA	gelatin methacryloyl
PBS	phosphate-buffered saline
PC	phase-contrast
RT	room temperature
SEM	standard error of the mean
VEGF-A	vascular endothelial growth factor A
